# Genetic Drivers of Plant Root Colonisation by the Biocontrol Agent *Pseudomonas protegens* Pf‐5

**DOI:** 10.1111/1758-2229.70179

**Published:** 2025-08-19

**Authors:** Timothy M. Ghaly, Belinda K. Fabian, Silas H. W. Vick, Christie Foster, Amy J. Asher, Karl A. Hassan, Liam D. H. Elbourne, Ian T. Paulsen, Sasha G. Tetu

**Affiliations:** ^1^ School of Natural Sciences Macquarie University Sydney Australia; ^2^ ARC Centre of Excellence in Synthetic Biology Sydney Australia; ^3^ Number 8 Bio Sydney Australia; ^4^ Ramaciotti Centre for Genomics University of New South Wales Sydney Australia; ^5^ School of Environmental and Life Sciences University of Newcastle Newcastle Australia

**Keywords:** biofilms, competitive colonisation, plant growth‐promoting bacteria, rhizoplane, transposon‐insertion sequencing

## Abstract

Plant growth‐promoting bacteria can confer a range of health benefits to plants, and are increasingly being used in agriculture as bioinoculants to enhance crop performance and prevent diseases. However, within complex rhizosphere communities, their success as bioinoculants depends heavily on their capacity to competitively colonise root systems. Here, we uncover genetic determinants of root colonisation by the biocontrol agent *Pseudomonas protegens* Pf‐5, known for its ability to suppress multiple plant diseases. Using Transposon‐Directed Insertion Site Sequencing (TraDIS), we systematically assayed the entire *P. protegens* Pf‐5 genome to determine genes involved in colonising the rhizoplane of two key agricultural crops, cotton (*n* = 153 Pf‐5 genes) and wheat (*n* = 110 Pf‐5 genes). We find a large overlap of 80 *P. protegens* Pf‐5 genes which are important for colonisation fitness in both plants, suggesting that these encode core traits linked to root colonisation. In‐depth functional annotation of these genes, leveraging both protein sequence and structure, reveals key traits that promote *P. protegens* Pf‐5 rhizoplane fitness, including biofilm formation, surface motility, nucleotide and amino acid biosynthesis, sugar catabolism, iron uptake, low‐oxygen growth, and stress response mechanisms. These findings can help guide future design and selection of microbial inoculants with improved capacity for competitive root colonisation.

## Graphical abstract

We highlight key traits that contribute to competitive root colonisation by *Pseudomonas protegens* Pf‐5, a biocontrol agent used to promote plant growth. These findings can help guide the design of microbial inoculants with improved capacity for competitive root colonisation.

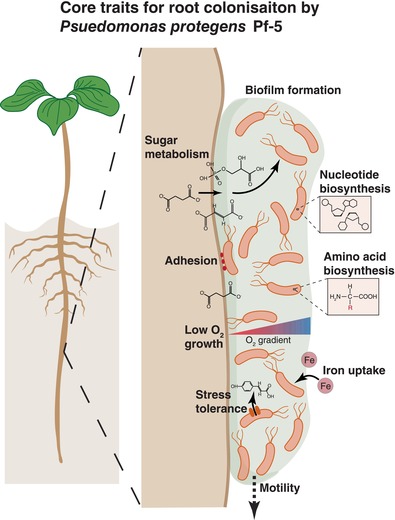

## 1 Introduction

Rhizosphere communities harbour diverse bacterial populations, which competitively colonise plant root surfaces and can significantly influence plant health. Within this ecosystem, plant growth‐promoting bacteria provide important benefits to plants, including improving nutrient and water uptake, alleviating abiotic stress, and suppressing plant pathogens and diseases (Trivedi et al. 2020). For this reason, there is growing interest in harnessing beneficial bacteria for improved plant performance, particularly in agricultural settings (de Vries et al. 2020; Shahwar et al. 2023). However, the success of using bacteria as plant growth‐promoting bioinoculants is largely dependent on their capacity for persistence on plant surfaces, providing a physical and metabolic niche (O'Callaghan et al. 2022). One key determining aspect is their ability to adhere to and colonise root surfaces. Understanding the genetic mechanisms that promote successful root colonisation is thus an essential step towards fully realising the potential of plant growth‐promoting bacteria in agriculture. In particular, using a trait‐based approach can help guide the selection or design of bioinoculants that are better able to colonise plant roots.

*Pseudomonas protegens* Pf‐5 is a plant‐associated bacterium recognised for its biocontrol activity, with the capacity to suppress a range of pathogens infecting important crops (Howell and Stipanovic 1979, 1980; Xu and Gross 1986; Kraus and Loper 1992; Pfender et al. 1993; Saravanan et al. 2013). Competitive root colonisation is a critical first step for biocontrol bacteria to successfully suppress plant pathogens. Previous studies have highlighted diverse genes in pseudomonads that assist in root colonisation (e.g., reviewed in (Lugtenberg et al. 2001; Zboralski and Filion 2020)). However, the genes involved in Pf‐5 root colonisation have not yet been fully explored.

Here, we used Transposon‐Directed Insertion Site Sequencing (TraDIS) to identify the *P. protegens* Pf‐5 genes associated with fitness in the rhizoplane of two important crops, cotton and wheat. TraDIS is a phenotypic characterisation technique that combines high‐density transposon insertion mutagenesis with high‐throughput DNA sequencing to functionally assay the entire genome of an organism (Langridge et al. 2009; Barquist et al. 2016). In this study, a population of *P. protegens* Pf‐5 transposon mutants, which contains knockouts of every non‐essential gene in the genome (Fabian et al. 2021), was used to inoculate cotton and wheat roots. By using transposon‐tagged DNA sequencing to determine mutants that were depleted or enriched in the rhizoplane, we identified Pf‐5 genes that are important for root colonisation. Our findings reveal key genes and pathways associated with enhanced *P. protegens* Pf‐5 fitness in the rhizoplane, highlighting beneficial traits related to biofilm formation, surface motility, nutrient uptake, sugar metabolism, and adaptive environmental response mechanisms.

## 2 Methods

### 2.1 Bacterial Strains

*Pseudomonas protegens* Pf‐5 (referred to hereafter as Pf‐5) used in this study was isolated from the soil of a cotton field in Texas, USA (Howell and Stipanovic 1979), and a complete genome sequence for this strain has been made available (Paulsen et al. 2005). For the present study, we used our previously constructed Pf‐5 Tn*5* transposon mutant library (Fabian et al. 2021). The Pf‐5 mutant library contains ~2560 unique transposon insertion sites spread evenly throughout the genome (~45 insertion sites per non‐essential gene) (Fabian et al. 2021).

### 2.2 Seed Surface Sterilisation and Bacterial Inoculation

Durum wheat ( *Triticum durum* ) and cotton ( *Gossypium hirsutum* ) seeds were surface sterilised with 80% v/v ethanol for 10 min, and then rinsed twice for 2 min with sterile de‐ionised water. Seeds were germinated on moistened cotton wool at 22°C and 28°C for wheat and cotton seeds, respectively. After 40 h, germinated seeds with a 1–2 cm radicle were inoculated with the Pf‐5 transposon mutant library by soaking in a bacterial inoculum for 1 h at 28°C without shaking. The inoculum comprised a mixture of three independent aliquots of the Pf‐5 transposon mutant library with a final density of 1 × 10^9^ CFU/mL. For plant growth controls, seeds underwent the same procedure but were soaked in phosphate buffered saline (PBS; Sigma Aldrich), instead of the bacterial inoculum.

### 2.3 Plant Growth Experiments

Plants were grown in sterile 500 mL flat‐bottomed screw cap lidded polypropylene sample jars (67 mm diameter × 150 mm height; Bacto Laboratories, Australia). Each jar contained 120 ± 1 g of autoclave‐sterilised substrate, composed of a 50:50 v/v mix of perlite (Brunnings, Australia) and washed sand (Dingo Cement, Australia), supplemented with 40 mL autoclaved quarter‐strength Hoagland's No. 2 Basal Salt Mixture (Sigma‐Aldrich, Australia). Since Hoagland's Solution (Hoagland and Arnon 1938) contains no carbon source, growth of Pf‐5 is dependent on the carbon compounds exuded from the plant roots. Five Pf‐5 mutant library‐inoculated seeds of the same species were planted in a single jar, and each jar was treated as a technical replicate. In total, 12 jars were used for each plant species. Six randomly selected jars were pooled to form one TraDIS replicate, resulting in two replicates per species.

The polypropylene jars containing the inoculated germinated seeds were placed in plant growth cabinets with 1000 μmol/m^2^/s of light at 22°C/16°C 12/12 h day/night cycles for wheat, and 30°C/22°C 12/12 h day/night cycles for cotton. Jar positions in the growth cabinets were randomised, and lids were regularly loosened and resealed to ensure adequate gas exchange.

For the Pf‐5 transposon library growth controls, nine replicate jars were set up consisting of the same substrate mix. These used sterile 50 mL tubes containing 12 g of the substrate, pre‐moistened with 4.5 mL quarter‐strength sterile Hoagland's Solution supplemented with 20 mM sodium succinate, and 300 μL of a 5 × 10^8^ CFUs/mL Pf‐5 transposon library suspension added to the surface of the substrate. Three randomly selected jars were pooled to form a single TraDIS control replicate.

Scanning electron microscopy was performed to confirm root colonisation by Pf‐5 (Figure S1). Briefly, this involved placing the selected root samples in a 3% glutaraldehyde solution for 24–48 h at room temperature, then washing three times in 0.1 M phosphate buffer. Samples were then dehydrated by sequential 10 min immersions in 30%, 50%, 70%, 80% and 90% ethanol solutions, two immersions in 100% ethanol, then one immersion in a 50:50 ethanol:hexamethyldisilazane solution, and finally, three immersions in 100% hexamethyldisilazane, before air drying for 24–48 h. Dehydrated samples were then gold sputter coated to 20 nm thickness and visualised with a JEOL JSM‐6480LV scanning electron microscope.

Plant growth controls were set up the same way, but did not have any added P‐5 transposon library. That is, control seeds underwent the same procedure but were soaked in phosphate buffered saline (PBS; Sigma Aldrich) instead of the Pf‐5 library inoculum. Five growth control seeds were planted into three negative control sample jars.

### 2.4 Recovery of *Pseudomonas protegens* Pf‐5 Mutant Library From Plant Roots

Pf‐5 library‐inoculated seedlings were harvested after six (wheat) and 7 days (cotton) of plant growth. Plants were removed from the bulk substrate and their roots were gently shaken in sterile de‐ionised water to remove larger sand and perlite particles. The roots were severed from the rest of the plant and placed in 15 mL tubes with 10 mL PBS with 0.05% Tween 20 (PBST) for wheat and 13 mL PBST for cotton. The roots were sonicated for 10 min (wheat) and 30 min (cotton) at 40 kHz, and then vortexed at maximum speed for 30 s (wheat) and 2 min (cotton). Sonicated roots with PBST were centrifuged at 4000 × *g* for 15 min to pellet the Pf‐5 cells detached from the root surface (rhizoplane mutant pools).

The Pf‐5 transposon library growth controls (i.e., in the absence of plant roots) had 40 mL PBST added to the perlite: sand mixture and sonicated for 20 min at 40 kHz; then vortexed at maximum speed for 30 s. The supernatant was removed from around the substrate and then centrifuged at 7830 rpm for 10 min to pellet the cells. Cell pellets from three technical replicates were combined to form each biological replicate (control mutant pools).

### 2.5 DNA Sequencing and Data Processing

DNA was extracted from the cells recovered from the surface of the cotton and wheat roots (rhizoplane mutant pools) and the cells recovered from the substrate with no plants (control mutant pools). The cells were lysed with an overnight incubation at 37°C with 100 mM EDTA and 10 mg/mL lysozyme (Sigma‐Aldrich), followed by a second overnight incubation at 56°C with 20 mg/mL Proteinase K (Sigma‐Aldrich). Genomic DNA was extracted from the cell lysate using the DNeasy Blood and Tissue Kit (Qiagen) as per the manufacturer's protocol. Genomic DNA from six technical replicate jars was combined to form each biological replicate (rhizoplane mutant pools).

Both biological replicates from the rhizoplane pools, and two randomly selected control pools, underwent Transposon‐Directed Insertion Site Sequencing (TraDIS). The DNA library preparation and sequencing were performed at the Ramaciotti Centre for Genomics (UNSW, Sydney, Australia) following previously described methods (Langridge et al. 2009; Barquist et al. 2016). The following sequencing primers were used: 5′ PCR primer—AATGATACGGCGACCACCGAGATCTACACATGATGATATATTTTTATCTTGTGCAATGTAACATC and 5′ Transposon sequencing primer—C*AGAGATTTTGAGACACAACGTGGCAGATGTGT*A. Sequencing was performed on an Illumina MiSeq platform to obtain 52 bp single‐end reads. The resulting reads were quality checked using FastQC v0.11.5 (Andrews 2010), and the transposon insertion sites were mapped to the Pf‐5 genome, and analysed using the Bio‐Tradis pipeline (Barquist et al. 2016). Following previously established parameters (Fabian et al. 2021, 2024; Vick et al. 2021), we allowed a 1 bp mismatch in the transposon tag, and excluded transposon insertions in the 3′ end of a gene. Reads with more than one mapping location were randomly mapped to a matching location to avoid repetitive elements artificially appearing significant. An average of 0.80 million reads was mapped to the Pf‐5 genome per replicate (Table S1). We validated the reproducibility between duplicates using linear regression of the gene insertion indexes, which showed a strong and significant correlation between all replicate pairs (*r* ^*2*^ > 0.88, *p* < 2.2e‐16; Figure S1). This high concordance and statistical significance indicate that the biological signals were highly reproducible between duplicates.

To assess differential frequencies of insertion sites between treatment and control mutant pools, we used the Bio‐Tradis script, *tradis_comparison R*, which applies edgeR (Robinson et al. 2010) to identify significant differences in read counts (Tables S2 and S3). Only genes with a log_2_‐fold change < −2 (i.e., fitness detrimental when function is lost) or > 2 (i.e., fitness beneficial when function is lost), and a FDR‐adjusted *p* value (*q*‐value) < 0.01 were used for further analysis (Figure S2, Table S4).

### 2.6 Gene Functional Annotations

Functional annotation of protein sequences was performed using eggNOG‐mapper v2 (Cantalapiedra et al. 2021) based on eggNOG v5 orthology data (Huerta‐Cepas et al. 2018). Annotation of amino acid biosynthesis pathways was performed using GapMind (Price et al. 2020). Proteins of unknown function, or those assigned only a broad‐level function, were additionally annotated based on their predicted protein structures. We used AlphaFold 3 (Abramson et al. 2024) for structure predictions, which were subject to structural homology searching with Foldseek (van Kempen et al. 2024) against the Protein Data Bank (Burley et al. 2023), the AlphaFold/SwissProt (Boeckmann et al. 2003; Varadi et al. 2023), and the AlphaFold/UniProt50 (Consortium 2022; Varadi et al. 2023) databases.

### 2.7 Cross‐Study Comparison of Genetic Drivers of *Pseudomonas* Root Colonisation

Genes associated with improved root colonisation among other Pseudomonads were obtained from previous transposon‐insertion sequencing studies (Cole et al. 2017; Sivakumar et al. 2019). Clusters of orthologous genes among the datasets were identified using OrthoVenn3 (Sun et al. 2023), employing the OrthoFinder program (Emms and Kelly 2019) for orthology inference with an *e*‐value threshold of 1e‐5 and an inflation value of 1.5 used for the Markov clustering algorithm (Van Dongen 2000).

## 3 Results and Discussion

**FIGURE 1:**
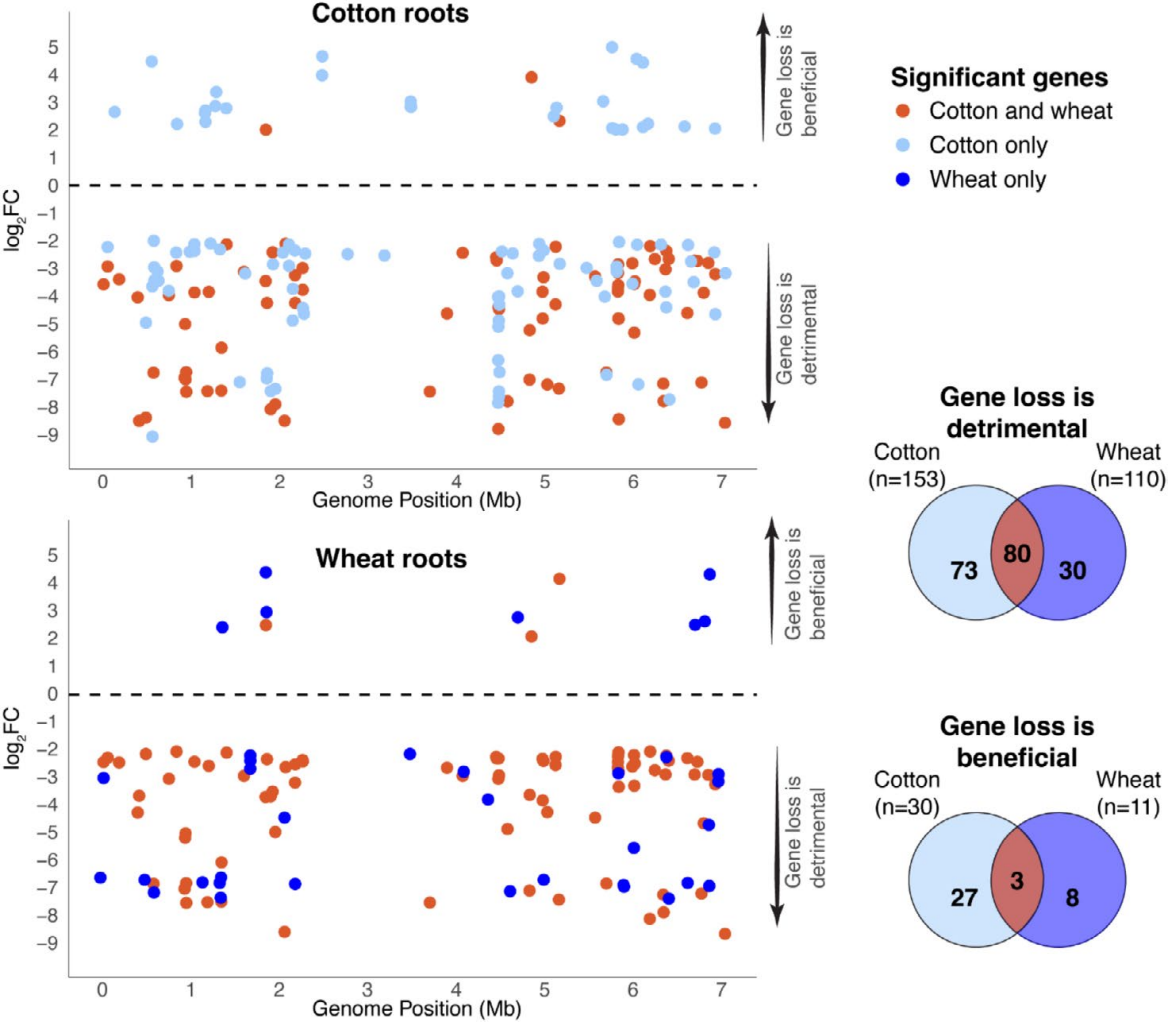
*Pseudomonas protegens* Pf‐5 genes associated with plant root colonisation fitness, as identified by TraDIS. Genome positions of Pf‐5 genes associated with cotton (top) and wheat (bottom) root colonisation. Only significant genes with a *q*‐value (*p*‐value adjusted for false discovery rate) < 0.01 and with a log_2_‐fold change < −2 or > 2 are shown. Points are coloured by plant type, where red indicates significant Pf‐5 genes associated with the colonisation of both plants; light blue are Pf‐5 genes significant for cotton root colonisation only; and dark blue are Pf‐5 genes significant for wheat root colonisation only. The respective numbers of genes for each are shown by the Venn diagrams. Note, a log_2_‐fold change < −2 indicates that gene loss of function was associated with decreased rhizoplane colonisation fitness, while genes with a log_2_‐fold change > 2 indicates that gene loss of function was associated with increased rhizoplane colonisation fitness. Pf‐5 genome positions are reported on the chromosome reoriented to begin at the *dnaA* gene (i.e., at the origin of replication).

We employed TraDIS using a *Pseudomonas protegens* Pf‐5 transposon mutant library, collectively containing knockouts of every non‐essential gene (Fabian et al. 2021), to identify the set of genes important for colonisation of cotton and wheat seedling roots. To identify Pf‐5 genes linked to rhizoplane fitness, cells recovered from the surface of plant roots were subjected to TraDIS sequencing, and the read frequency of transposon insertions in each gene was compared with that observed in the control groups (i.e., cells collected from the substrate in the absence of roots). These experiments identified 153 Pf‐5 genes for which loss of function was associated with decreased cotton rhizoplane colonisation fitness and 110 associated with decreased wheat root colonisation fitness, with 80 of these in common to both plants (Figure 1). Additionally, we identified a set of 30 genes for which loss of function was associated with improved cotton root colonisation and 11 genes for wheat roots, with 3 genes in common to both plants (Figure 1). We note that there are fewer root colonisation‐associated genes located between the 2.5 and 4 Mb region of the Pf‐5 genome (Figure 1). This region, which spans the terminus of replication, is also depleted in Pf‐5 essential genes (Fabian et al. 2021). Generally, essential genes required for replication and growth are organised closer to the origin of replication, with the terminus more variable and subject to higher rates of horizontal gene transfer and genetic rearrangements (Silby et al. 2009). Similarly, conserved genes involved in important lifestyle traits (such as root colonisation in Pf‐5) might also be expected to occur less frequently in the terminus region, as observed here (Figure 1).

**FIGURE 2:**
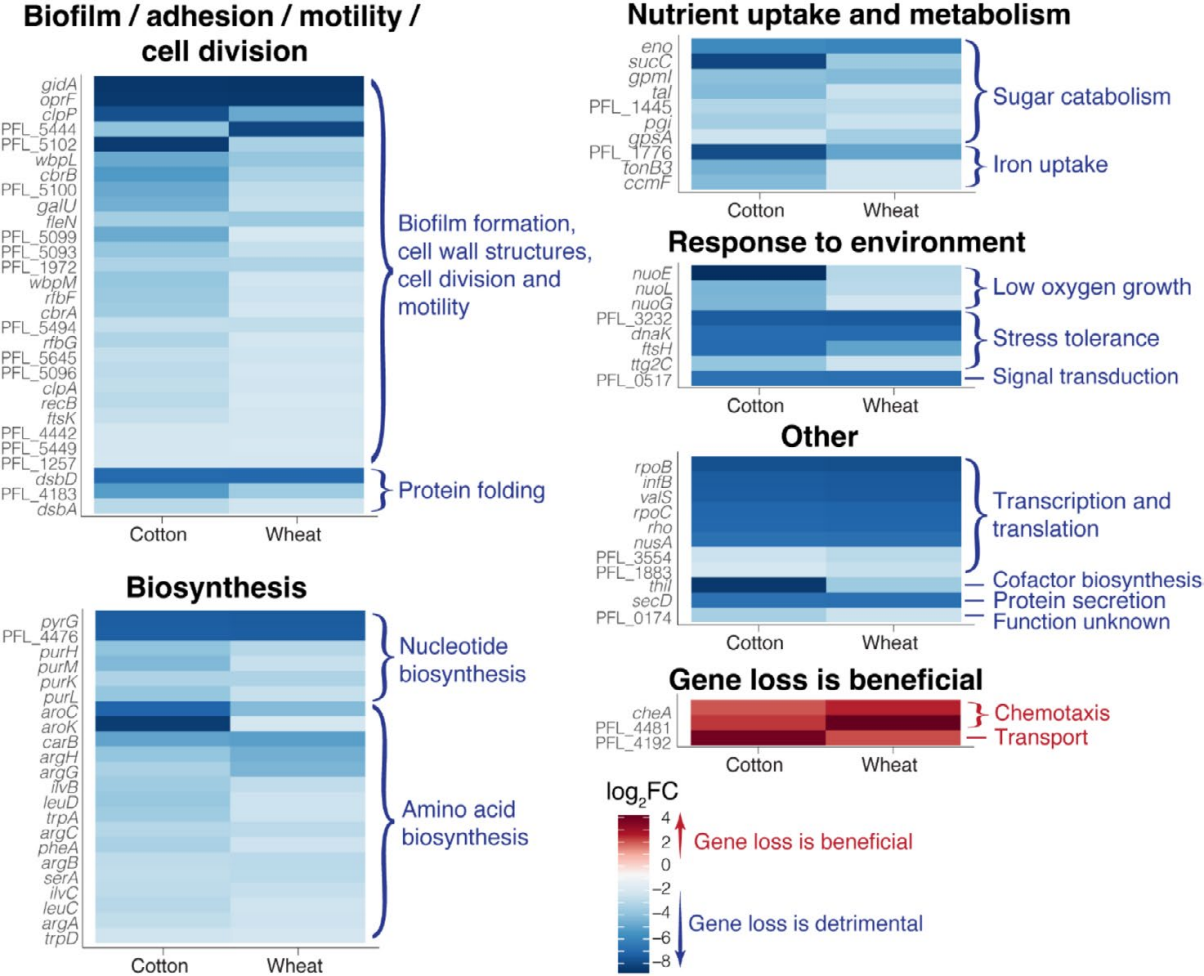
Heatmap showing log_2_‐fold change of *Pseudomonas protegens* Pf‐5 mutants associated with root colonisation. Blue colours indicate genes for which loss of function is associated with a reduction in root colonisation fitness for both cotton and wheat (with respective mutants having a log_2_‐FC < −2), while red colours indicate genes for which loss of function is associated with improved cotton and wheat root colonisation (with respective mutants having a log_2_‐FC > 2). Note, that while several genes likely span multiple functional categories (as discussed in the Results), for simplicity, here they are presented under just one category.

The set of 80 genes that were identified as being important for colonisation of both cotton and wheat roots likely encode functions that are core to Pf‐5 rhizoplane colonisation. The degree of overlap in genes associated with the tested plant species was of particular interest given the physiological difference in the root systems of cotton, a dicot with a taproot system, and wheat, a monocot with a fibrous root network. We performed in‐depth functional annotation to assign functions to 79 out of 80 of these genes, made possible by combining protein sequence and structure homology detection methods. Together, the genes spanned diverse functional categories, with many involved in promoting a biofilm lifestyle, surface motility, nutrient uptake, and environmental response mechanisms (Figure 2). All of these traits are thus likely contributing to Pf‐5 rhizoplane fitness.

### 3.1 Genes Involved in Biofilm Formation, Cell Adhesion and Motility

Of the 80 significant Pf‐5 genes important for root colonisation of both plants, a large set (*n* = 29) are known to contribute to bacterial biofilm formation, surface adhesion, and/or motility (Figure 2). Some are directly involved in these processes, including *oprF*, encoding an outer membrane porin needed for binding and adhesion to host cells (Chevalier et al. 2017), and *fleN*, encoding a master regulator of both flagellar motility and biofilm formation genes (Torres‐Sánchez et al. 2023). There were also various genes involved in the synthesis and maintenance of cell envelope structures, which are important for surface attachment (Hori and Matsumoto 2010). These include lipopolysaccharide biosynthesis genes: *wbpL*, *wbpM*, *rfbF*, *rfbG*, *galU*, PFL_5096 (encoding RfbH), PFL_5100 (encoding RfbV), PFL_5093 (encoding a CDP‐6‐deoxy‐L‐threo‐D‐glycero‐4‐hexulose‐3‐dehydrase reductase), PFL_5494 (encoding an O‐antigen transporter), PFL_5099 (encoding an O‐unit flippase), and PFL_5102 (encoding a putative glycosyltransferase); peptidoglycan recycling genes: PFL_5645 (encoding an N‐acetylmuramate/N‐acetylglucosamine kinase), PFL_5444 (encoding a putative murein peptide carboxypeptidase), and PFL_1257 (encoding an anhydromuropeptide permease); as well as a cell envelope integrity gene: PFL_4442 (encoding an EipB‐like outer membrane lipoprotein). All these genes, which are involved in synthesising and modulating cell envelope structures, likely play an important role in Pf‐5 adhesion to root surfaces.

Other genes in this set are likely to be important for rhizosphere colonisation due to indirect effects on biofilm formation and motility. This included *gidA*, which encodes a tRNA modification enzyme that modifies uridine at the wobble position of tRNAs (Meyer et al. 2008), and which exhibited the largest fold change for both cotton and wheat colonisation assays (Figure 2). Among Pseudomonads, GidA‐driven tRNA modifications drive significant proteomic shifts, influencing a diverse set of traits, including biofilm formation and swarming motility (i.e., biofilm spread via surface migration) (Kinscherf and Willis 2002; Srimahaeak et al. 2023; Krueger et al. 2024). Disruption of *gidA* in *Pseudomonas aeruginosa* significantly reduces the production of flagellar and biofilm formation proteins (Srimahaeak et al. 2023), suggesting that its likely role in Pf‐5 root colonisation is to promote biofilm establishment and surface motility.

The *clpP* gene, encoding a serine protease, was also important for colonisation of roots of both plants (Figure 2). ClpP is associated with multiple mechanisms that influence biofilm formation and motility. For example, in *Pseudomonas fluorescens* SS101, ClpP, together with the chaperone ClpA (also found here to be important for rhizoplane colonisation; Figure 2), regulates the biosynthesis of the cyclic lipopeptide massetolide A (Song et al. 2015), which in turn is essential for swarming motility and biofilm formation (Bruijn et al. 2008). Additionally, the ClpAP complex interacts with DnaK (likewise found here to be involved in rhizoplane fitness; Figure 2) to also influence cyclic lipopeptide biosynthesis in *Pseudomonas putida* and *P. fluorescens* (Dubern et al. 2005; Song et al. 2015). Cyclic lipopeptide production has been shown to promote *Pseudomonas* plant colonisation due to its biosurfactant activity, playing an important role in host surface attachment and biofilm formation (Nielsen et al. 2005; Tran et al. 2007). Pf‐5 produces the cyclic lipopeptide orfamide A (Gross et al. 2007), which is essential for surface motility (D'aes et al. 2014; Ma et al. 2016) in a manner analogous to massetolide A in related *Pseudomonas* species. While the ClpAP protease has been shown to regulate massetolide biosynthesis in Pseudomonads, its potential role in modulating orfamide production in Pf‐5 remains unexplored. The ClpP protease might also influence motility through its role in the degradation of the RpoS sigma factor (a transcriptional regulator), which is delivered to ClpP by the response regulator, RssB (Rodríguez‐Martínez et al. 2023) (*rssB*, annotated as PFL_1972, is also detrimental when lost; Figure 2). ClpP‐mediated degradation of RpoS significantly increases swimming and swarming ability in *Serratia marcescens* (Qin et al. 2020). In support of this, loss of *rpoS* function was found here to be associated with improved root colonisation in both wheat (log_2_FC = 2.13) and cotton (log_2_FC = 1.82), suggesting that depletion of cellular RpoS promotes root colonisation via enhanced motility.

Other biofilm, attachment, and/or motility‐associated genes important for both cotton and wheat root colonisation were involved in signalling, cell division, and protein folding (Figure 2). These include the genes encoding the two‐component sensor histidine kinase, CbrA, and response regulator, CbrB, which make up a *Pseudomonas*‐specific signal transduction system. These genes were also found to be important in a rhizospheric *P. aeruginosa* strain, with their inactivation driving dysregulation of motility and biofilm formation genes, leading to a 10‐fold decrease in corn root colonisation (Sivakumar et al. 2021). The cell division genes were *ftsK*, encoding a DNA translocase, and *rlpA*, encoding a lipoprotein, which together aid chromosomal segregation and daughter cell separation (Jorgenson et al. 2014; Berezuk et al. 2018). Optimal cell division is essential for efficient population surface migration via swarming motility (Wei and Lai 2006), likely contributing here to Pf‐5 rhizoplane fitness. Additionally, we found *recB* to be important for root colonisation. RecB is involved in the repair of double‐stranded DNA breaks, which increase in frequency during cell division, particularly in the terminus region of the chromosome (Sinha et al. 2017). Its inactivation in *E. coli* drives the loss of terminus DNA (Sinha et al. 2017), indicating the importance of RecB for genome integrity during periods of increased cell division. RecB‐mediated repair has also been reported to help buffer against oxidative DNA damage that can be caused by root exudates, and is also required by the biocontrol agent *Pseudomonas pseudoalcaligenes* for rhizosphere colonisation (Pliego et al. 2019). We also note several significant genes involved in protein folding, comprising *dsbA*, *dsbD*, and PFL_4183, which all encode thiol: disulfide interchange proteins. These proteins influence the development of cell surface protein structures, including fimbriae (pili) and flagella (Dailey and Berg 1993; Shevchik et al. 1995; Kloek et al. 2000), important for surface attachment and motility. Indeed, disruption of *dsbA* in *P. fluorescens* Q8r1–96 reduces its competitive colonisation of wheat roots (Mavrodi et al. 2006).

**FIGURE 3:**
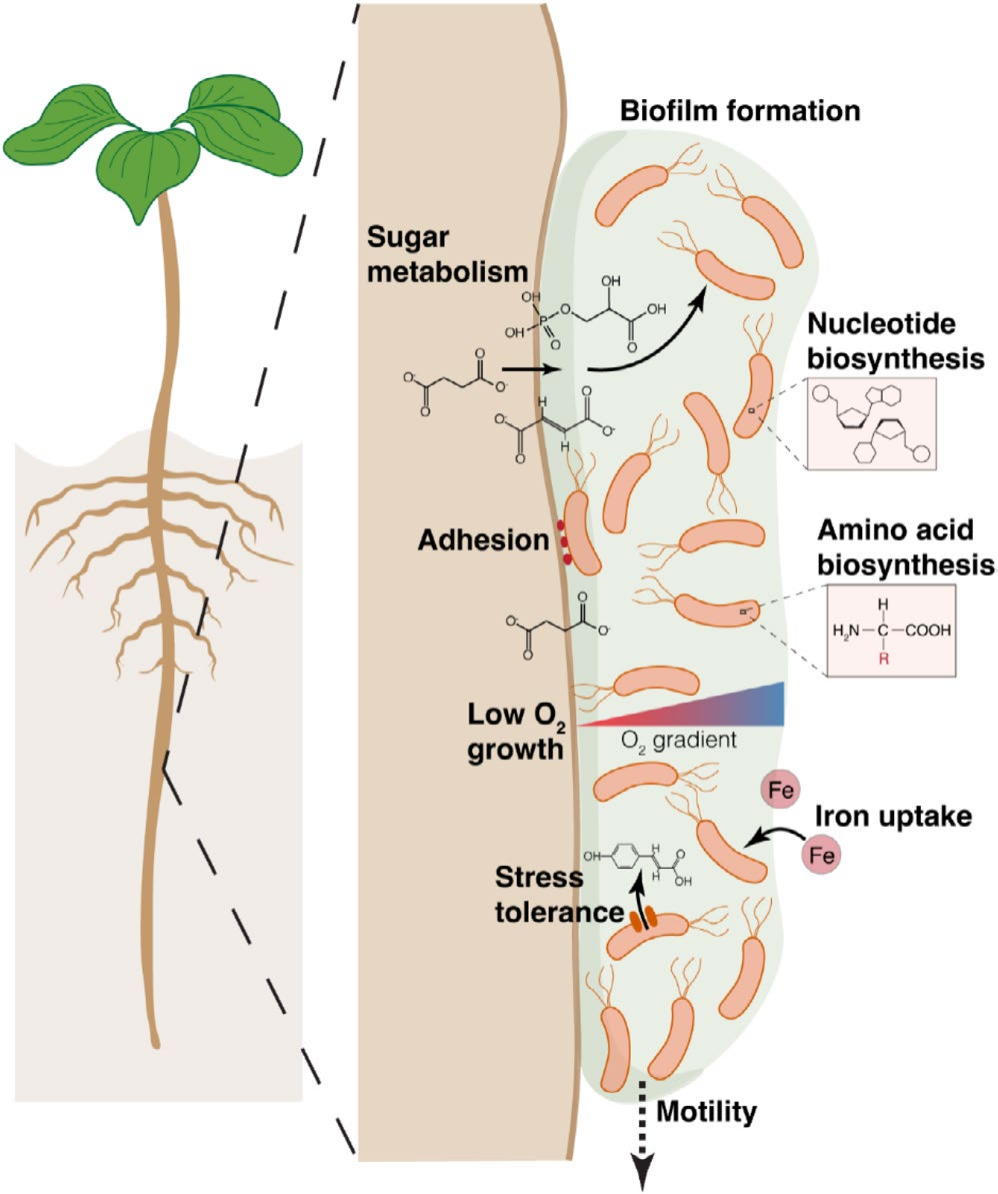
Conceptual diagram of *Pseudomonas protegens* Pf‐5 traits important for root colonisation. Functional annotation of genes implicated in Pf‐5 colonisation of both cotton and wheat roots, as identified by TraDIS, was used to identify traits likely important to root colonisation. These largely consist of biofilm formation, cell adhesion, motility, amino acid and nucleotide biosynthesis, sugar metabolism, iron uptake, low oxygen growth, and stress tolerance.

Together, this diverse set of genes is associated with Pf‐5 fitness in the rhizoplane. Their known functions indicate that surface attachment, biofilm formation, cell division, and surface migration are some of the key traits identified here as important for Pf‐5 root colonisation fitness (Figure 3).

### 3.2 Amino Acid and Nucleotide Biosynthesis Genes

We identified a large number of amino acid biosynthesis genes involved in Pf‐5 root colonisation (Figure 2). These made up 20% (16 out of 80) of the Pf‐5 genes important for colonising the roots of both plants, representing a significant enrichment (*χ* ^2^ test; *p* < 0.00001) relative to the whole Pf‐5 genome (3.18%). These included the aromatic biosynthesis genes *aroC*, *aroK*, *pheA*, *trpA*, *trpD*, which lead to the formation of tryptophan, tyrosine, and phenylalanine; as well as genes involved in the synthesis of non‐aromatic amino acids, including arginine (*argA*, *argB*, *argC*, *argG*, *argH*, *carB*), isoleucine (*ilvB*, *ilvC*, *leuC*, *leuD*), and cysteine (*serA*). Despite amino acids being major components of plant root exudates, their biosynthesis is still evidently important for efficient Pf‐5 root colonisation. This has been previously reported for rhizospheric *Pseudomonas* strains. For example, *P. fluorescens* WCS365 mutants unable to synthesise either leucine, arginine, histidine, isoleucine, or tryptophan are incapable of colonising tomato roots (Simons et al. 1997), and in *P. aeruginosa* , tryptophan, arginine, cysteine, and leucine biosynthesis genes are needed for successful corn root colonisation (Sivakumar et al. 2019). Although a mechanistic role has yet to be established, we hypothesise that the increased capacity for amino acid production may aid root colonisation by improving the formation of a robust biofilm matrix. Proteins are a major structural extracellular component of biofilms, and amino acid biosynthesis enzymes are essential for the formation and maintenance of biofilms across diverse bacterial taxa (Sriramulu et al. 2005; Losensky et al. 2017; Mohammed et al. 2017; Lu et al. 2019; Suryaletha et al. 2019; Barrientos‐Moreno et al. 2020). Their significance for root colonisation may therefore be due to the increased demand for protein production needed for biofilms.

We also note that several additional significant rhizoplane colonisation genes are involved in nucleotide biosynthesis (e.g., *purH*, *purK*, *purl*, *purM*, *pyrG*, and PFL_4476, encoding a ribonucleoside‐diphosphate reductase), which have also been shown to be important for biofilm formation. For example, the disruption of nucleotide biosynthesises in *P. fluorescens* Pf0‐1 reduces biofilm formation and leads to reduced cell size of surface‐attached, but not planktonic, cells (Yoshioka and Newell 2016). Beyond biofilm formation, efficient biosynthesis of both nucleotides and amino acids is also important for increased rates of DNA replication and cell division that occur during population growth and surface migration. Interestingly, both amino acid and nucleic acid biosynthesis are also critical for plant pathogenesis by *Xanthomonas hortorum* (Morinière et al. 2022), suggesting that these are universal functions required for plant colonisation by both pathogens and mutualists.

### 3.3 Nutrient Uptake and Metabolism Genes

The loss of function of several central carbon metabolic genes involved in sugar catabolism was also observed to be detrimental to Pf‐5 root colonisation (Figure 2). These included genes involved in glycolysis: *gpmI*, *eno*, *pgi*, *gpsA*; the pentose phosphate pathway: *tal*; and the TCA cycle: *sucC* and PFL_1445 (encoding the FAD assembly factor SdhE, which facilitates the incorporation of FAD into the enzymes succinate dehydrogenase and fumarate reductase (McNeil et al. 2014)). These central carbon metabolic genes are necessary for the catabolism of different sugars (e.g., glucose, fructose, sucrose) and organic acids (e.g., citrate, malate, oxalate) found in both cotton and wheat root exudate (Kumar et al. 2007). We also identified several genes involved in siderophore‐mediated iron uptake that are important for Pf‐5 root colonisation: *tonB* and PFL_1776 (encoding ExbB1). Iron is limiting in the rhizosphere since bacteria must compete for iron with the plant (Lemanceau et al. 2009). This makes iron scavenging mechanisms particularly important for rhizobacteria. We also identified *ccmF* as important for Pf‐5 root colonisation. While *ccmF* has been implicated in siderophore biosynthesis, and thus is important for iron scavenging, the impacts of its disruption on siderophore production are variable among *Pseudomonas* species (Baert et al. 2008). Its disruption can have pleiotropic effects, extending beyond siderophore production, including reduced growth and motility (Baert et al. 2008), all of which are likely to be relevant to plant colonisation.

### 3.4 Genes Involved in Pf‐5 Response to Environmental Conditions

The loss of several subunits of the NADH‐Quinone Oxidoreductase (Nuo) was detrimental to Pf‐5 root colonisation (Figure 2). The Nuo complex is essential for anaerobic growth in *Pseudomonas* spp. (Camacho Carvajal et al. 2002; Torres et al. 2019). For example, Nuo is required by *P. fluorescens* WCS365 for competitive root colonisation, where its expression is activated in the tomato rhizosphere and increases with declining oxygen concentrations (Camacho Carvajal et al. 2002). The capacity for low‐oxygen growth is particularly important in the rhizoplane (since plant roots can increase the anaerobic volume of the soil (Lecomte et al. 2018)) and within biofilms more generally, which are characterised by steep oxygen gradients due to incomplete penetration and rapid respiration of oxygen (Stewart et al. 2016; Klementiev et al. 2020).

We also identified genes involved in tolerance to toxic compounds. One of these, *ttg2C*, encodes an efflux pump associated with stress tolerance. In *Pseudomonas putida* , Ttg2C provides resistance to *p*‐coumaric acid (Calero et al. 2018), which is one of the major phenolic compounds in plants, including cotton (Kouakou et al. 2007) and wheat (Hernández et al. 2011). In addition, we identified the gene PFL_3232, which encodes a predicted structure highly similar to that of the quinone oxidoreductase in *P. syringae* (PDB accession 3JYL; TM‐Score: 0.88). In *P. syringae* , this enzyme breaks down cytotoxic quinones (Pan et al. 2009), which are known root exudates (Walker et al. 2003).

We also identified stress response genes, *ftsH* and *dnaK*, as being associated with Pf‐5 rhizoplane fitness. FtsH is an inner membrane–anchored metalloprotease. In *P. aeruginosa* , FtsH promotes survival during proteostatic stress caused by environmental conditions, such as nutrient starvation, alkaline pH, or heat shock (Basta et al. 2020). DnaK is a stress‐response chaperone protein. Beyond its role in stress response, DnaK is also important for motility. As mentioned previously, DnaK is implicated in cyclic lipopeptide biosynthesis, which is associated with motility and biofilm formation in *Pseudomonas* spp. (Dubern et al. 2005; Song et al. 2015). DnaK is also involved in flagellar formation in *P. aeruginosa* (Acuña et al. 2015) and surface motility in Cyanobacteria (McDonald et al. 2022). Thus, DnaK probably plays a broader role in Pf‐5 root colonisation, facilitating motility as well as stress response regulation.

The Pf‐5 gene PFL_0517, encoding a serine/threonine protein kinase, was highly significant for both plant colonisation assays (Figure 2). These enzymes regulate signal transduction pathways via protein phosphorylation at serine and threonine residues. This functionally activates its protein substrate and/or triggers the transfer of the phosphate group to downstream proteins, permitting a cascade of signal transduction (Canova and Molle 2014). This process allows bacteria to readily respond to environmental conditions through rapid signal‐response reactions (Canova and Molle 2014). Such a trait is likely allowing an adaptive Pf‐5 response to a physical or biochemical cue in the rhizoplane.

### 3.5 Pf‐5 Genes for Which Loss of Function Appears Beneficial to Rhizoplane Colonisation

We identified three genes for which loss of function was associated with enhanced Pf‐5 colonisation of both cotton and wheat roots (log_2_‐FC > 2), implying that these genes have a fitness cost during Pf‐5 rhizoplane growth (Figure 2). Surprisingly, two of the three were chemotaxis genes: *cheA* and PFL_4481 (encoding CheR). Chemotaxis is generally considered an important trait for root colonisation, as it allows bacteria to detect and move towards root exudates, which exist as chemical gradients extending from root surfaces (Keegstra et al. 2022). It is possible, however, that once established on the root surface, chemotaxis may provide little benefit relative to its energetic cost. In this context, inhibition of chemotaxis‐driven motility, whilst still maintaining the capacity for surface migration, may be more energy efficient, and thus provide a greater fitness advantage.

The third gene, PFL_4192, encodes a predicted structure that shares greatest homology with the crystal structure of an amino‐acid uptake ABC transporter from *Thermus thermophilus* (PDB accession 3vv5; TM‐Score: 0.73). *T. thermophilus* is hypersensitive to the lysine analogue, *S*‐2‐aminoethyl‐cysteine (AEC), and inactivation of this ABC transporter confers AEC resistance by preventing its uptake (Kanemaru et al. 2013). Thus, Pf‐5 mutants lacking this ABC transporter might have decreased susceptibility to cytotoxic analogues of amino acids exuded by plant roots. For example, in *P. fluorescens* WCS365, increased uptake of several root exudates by ABC transport systems can trigger bacteriostatic effects, hindering capacity for competitive root colonisation (Kuiper et al. 2001).

### 3.6 Pf‐5 Genes Involved in Plant‐Specific Colonisation

We identified subsets of genes that were significantly associated with root colonisation fitness for only one of the two plant types (Figure 1). There were more genes that, when function was lost, resulted in decreased root colonisation fitness for cotton only (*n* = 73), compared to wheat only (*n* = 30). This may be because Pf‐5 was originally isolated from the rhizosphere of cotton (Howell and Stipanovic 1979), and thus may have an accessory genome better adapted for cotton root colonisation. The beneficial genes specific to cotton colonisation are largely involved in the same processes that we identified above to be important for the colonisation of both plants, including sugar metabolism, iron transport, amino acid biosynthesis, as well as genes encoding additional subunits of the Nuo complex, permitting low‐oxygen growth (Table S2).

A large number of the genes (18 out of 30) for which loss of function significantly reduced root colonisation fitness for wheat also showed a trend for reduced cotton root colonisation but fell below the cutoffs used in this work exhibiting either a log_2_‐FC < −2, but a *q*‐value > 0.01 (although these had *q*‐values < 0.05, or a log_2_‐FC less than −1.9, but still greater than −2) (Figure 1, Table S4). These were largely comprised of genes involved in replication, transcription, and translation, lipid metabolism, and cell wall synthesis. The remaining genes important for wheat‐only root colonisation were involved in sugar metabolism, amino acid biosynthesis, signal transduction, motility, and iron uptake (Table S4).

### 3.7 Cross‐Study Comparison of Root Colonisation‐Associated Genes in *Pseudomonas*

**FIGURE 4:**
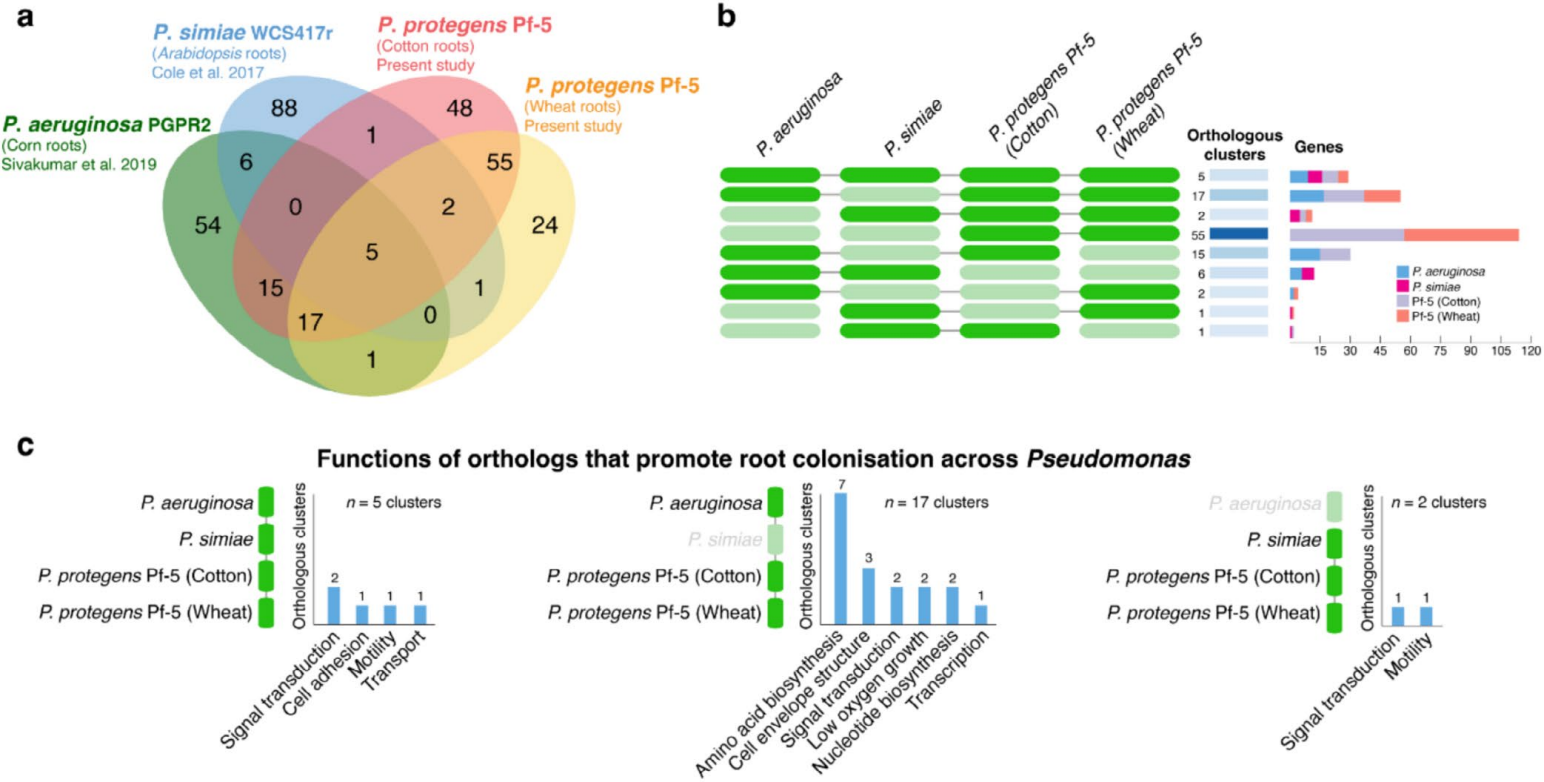
Cross‐study comparison of root colonisation‐associated genes in *Pseudomonas*. (a) A Venn diagram showing the overlap in clusters of orthologous genes identified to be putatively beneficial for root colonisation among *Pseudomonas* spp. (b) Overlap in orthologous cluster and gene counts between the different transposon‐insertion sequencing dataset comparisons. (c) Functional annotations of orthologous clusters common to Pf‐5 (cotton and wheat), *P. aeruginosa* and *P. simiae* (left); Pf‐5 (cotton and wheat) and *P. aeruginosa* only (middle); and Pf‐5 (cotton and wheat) and *P. simiae* only (right).

To identify conserved genetic determinants of root colonisation, we analysed overlapping genes linked to improved colonisation fitness across different *Pseudomonas* species and plant host systems (Figure 4, Table S5). This involved comparative integration of transposon‐insertion sequencing datasets from *P. simiae* WCS417r colonising *Arabidopsis thaliana* (Cole et al. 2017), and *P. aeruginosa* PGPR2 colonising corn roots (Sivakumar et al. 2019). Notably, a core set of five orthologous genes emerged as important for root colonisation in *P. protegens* Pf‐5 (cotton and wheat), *P. simiae* , and *P. aeruginosa* (Figure 4a,b). These genes, implicated in signal transduction, cell adhesion, motility regulation, and ABC transporter activity (Figure 4c; Table S5), may represent universally important *Pseudomonas* rhizoplane colonisation mechanisms.

PF‐5 shared a larger overlap of putative root colonisation‐promoting genes with *P. aeruginosa* than *P. simiae* (Figure 4a,b). We identified 17 orthologous clusters associated with improved root colonisation for PF‐5 (cotton and wheat) and *P. aeruginosa* (corn). These were involved in amino acid/nucleotide biosynthesis, cell envelope structure, low‐oxygen adaptation, and transcriptional regulation (Figure 4c). Additionally, PF‐5 (cotton and wheat) and *P. simiae* (*Arabidopsis*) shared genes associated with signal transduction and motility (Figure 4c). These shared genes highlight their conserved functional importance for root colonisation across *Pseudomonas* species and divergent plant host systems.

## 4 Conclusion

Identifying the suite of genes and processes which contribute to root colonisation fitness in plant growth‐promoting bacteria is a key undertaking, not only to better understand plant‐microbe interactions, but also to help facilitate plant microbiome interventions for improved plant performance. The capacity for effective root colonisation and subsequent growth in this niche is essential for rhizoplane persistence, and thus should be a key consideration for the design and selection of successful microbial inoculants. Here, using TraDIS, we identified diverse sets of genes that are important for the fitness of the plant growth‐promoting strain, *P. protegens* Pf‐5, in the rhizosphere of cotton (*n* = 153) and wheat (*n* = 110). We found a large overlap of 80 genes important for the colonisation of both plants, suggesting that these are linked to core mechanisms involved in Pf‐5 root colonisation (Figure 3). In‐depth functional annotation of these genes indicates that the key traits for Pf‐5 root colonisation include surface attachment and migration, biofilm formation, nutrient uptake and metabolism (particularly sugars and iron), low‐oxygen growth, and stress response mechanisms. While many of the genes identified here have been linked to rhizoplane/rhizosphere fitness in other pseudomonads previously, this approach extends our understanding, identifying a number of additional genes hypothesised to be important. These findings highlight the benefit of applying techniques such as TraDIS to expand our understanding of the traits contributing to rhizoplane fitness in plant growth‐promoting (PGP) bacteria such as Pf‐5. Such knowledge has the potential to help guide the development of more effective application of microbe‐based strategies, such as field application of PGP bacteria, for improved crop production.

## Author Contributions

**Timothy M. Ghaly:** formal analysis, writing – review and editing, writing – original draft, visualization, methodology. **Belinda K. Fabian:** methodology, writing – review and editing, conceptualization, formal analysis. **Silas H. W. Vick:** methodology, writing – review and editing. **Christie Foster:** methodology, writing – review and editing. **Amy J. Asher:** writing – review and editing, methodology. **Karl A. Hassan:** conceptualization, funding acquisition, writing – review and editing, methodology. **Liam D. H. Elbourne:** methodology, formal analysis, writing – review and editing. **Ian T. Paulsen:** conceptualization, funding acquisition, writing – review and editing, methodology. **Sasha G. Tetu:** conceptualization, funding acquisition, writing – review and editing, methodology.

## Conflicts of Interest

The authors declare no conflicts of interest.

## Supporting information


**Figure S1.** *Pseudomonas protegens* Pf‐5 root colonisation and TraDIS duplicate reproducibility. (a) Scanning electron microscopy images of Pf‐5 colonisation of cotton roots (top) and wheat roots (bottom). (b) Correlation of gene insertion indexes for the duplicate TraDIS assays for cotton (top) and wheat (bottom). Insertion indices represent the number of transposon insertion sites for a given gene normalised by gene length.
**Figure S2.** Differential frequencies of all *Pseudomonas protegens* Pf‐5 gene insertions. Volcano plots show log_2_ fold change and −log_10_ *q*‐values for root colonisation assays of a, cotton, and b, wheat, relative to the control. Points in dark blue have a significant log_2_‐fold change < −2, or > 2, and a *q*‐value < 0.01.

**Table S1.** TraDIS metrics from Bio‐Tradis pipeline analysis.
**Table S2.** Differential frequencies of *Pseudomonas protegens* Pf‐5 gene insertions between cotton root colonisation and control mutant pools.
**Table S3.** Differential frequencies of *Pseudomonas protegens* Pf‐5 gene insertions between wheat root colonisation and control mutant pools.
**Table S4.** Significant *Pseudomonas protegens* Pf‐5 genes associated with cotton and/or wheat root colonisation.
**Table S5.** Cross‐study comparison of root colonisation‐associated genes in *Pseudomonas*.

## Data Availability

The TraDIS sequence data generated in this study are openly available from figshare at https://doi.org/10.6084/m9.figshare.28225406.v1.

## References

[emi470179-bib-0001] Abramson, J. , J. Adler , J. Dunger , et al. 2024. “Accurate Structure Prediction of Biomolecular Interactions With AlphaFold 3.” Nature 630: 493–500. 38718835 10.1038/s41586-024-07487-w PMC11168924

[emi470179-bib-0002] Acuña, J. M. B.‐d. , G. Molinari , M. Rohde , et al. 2015. “A Periplasmic Complex of the Nitrite Reductase NirS, the Chaperone DnaK, and the Flagellum Protein FliC Is Essential for Flagellum Assembly and Motility in *Pseudomonas aeruginosa* .” Journal of Bacteriology 197: 3066–3075. 26170416 10.1128/JB.00415-15 PMC4560289

[emi470179-bib-0003] Andrews, S. 2010. “FastQC: A Quality Control Tool for High Throughput Sequence Data. In: Cambridge, United Kingdom.”

[emi470179-bib-0004] Baert, B. , C. Baysse , S. Matthijs , and P. Cornelis . 2008. “Multiple Phenotypic Alterations Caused by a c‐Type Cytochrome Maturation *ccmC* Gene Mutation in *Pseudomonas aeruginosa* .” Microbiology 154: 127–138. 18174132 10.1099/mic.0.2007/008268-0

[emi470179-bib-0005] Barquist, L. , M. Mayho , C. Cummins , et al. 2016. “The TraDIS Toolkit: Sequencing and Analysis for Dense Transposon Mutant Libraries.” Bioinformatics 32: 1109–1111. 26794317 10.1093/bioinformatics/btw022 PMC4896371

[emi470179-bib-0006] Barrientos‐Moreno, L. , M. A. Molina‐Henares , M. I. Ramos‐González , and M. Espinosa‐Urgel . 2020. “Arginine as an Environmental and Metabolic Cue for Cyclic Diguanylate Signalling and Biofilm Formation in *Pseudomonas putida* .” Scientific Reports 10: 13623. 32788689 10.1038/s41598-020-70675-x PMC7423604

[emi470179-bib-0007] Basta, D. W. , D. Angeles‐Albores , M. A. Spero , J. A. Ciemniecki , and D. K. Newman . 2020. “Heat‐Shock Proteases Promote Survival of *Pseudomonas aeruginosa* During Growth Arrest.” Proceedings of the National Academy of Sciences of the United States of America 117: 4358–4367. 32029587 10.1073/pnas.1912082117 PMC7049150

[emi470179-bib-0008] Berezuk, A. M. , S. Glavota , E. J. Roach , M. C. Goodyear , J. R. Krieger , and C. M. Khursigara . 2018. “Outer Membrane Lipoprotein RlpA Is a Novel Periplasmic Interaction Partner of the Cell Division Protein FtsK in *Escherichia coli* .” Scientific Reports 8: 12933. 30154462 10.1038/s41598-018-30979-5 PMC6113214

[emi470179-bib-0009] Boeckmann, B. , A. Bairoch , R. Apweiler , et al. 2003. “The SWISS‐PROT Protein Knowledgebase and Its Supplement TrEMBL in 2003.” Nucleic Acids Research 31: 365–370. 12520024 10.1093/nar/gkg095 PMC165542

[emi470179-bib-0010] Bruijn, I. D. , M. J. D. D. Kock , P. D. Waard , T. A. V. Beek , and J. M. Raaijmakers . 2008. “Massetolide A Biosynthesis in *Pseudomonas fluorescens* .” Journal of Bacteriology 190: 2777–2789. 17993540 10.1128/JB.01563-07 PMC2293227

[emi470179-bib-0011] Burley, S. K. , C. Bhikadiya , C. Bi , et al. 2023. “RCSB Protein Data Bank (RCSB. Org): Delivery of Experimentally‐Determined PDB Structures Alongside One Million Computed Structure Models of Proteins From Artificial Intelligence/Machine Learning.” Nucleic Acids Research 51: D488–D508. 36420884 10.1093/nar/gkac1077 PMC9825554

[emi470179-bib-0012] Calero, P. , S. I. Jensen , K. Bojanovič , R. M. Lennen , A. Koza , and A. T. Nielsen . 2018. “Genome‐Wide Identification of Tolerance Mechanisms Toward p‐Coumaric Acid in *Pseudomonas putida* .” Biotechnology and Bioengineering 115: 762–774. 29131301 10.1002/bit.26495 PMC5814926

[emi470179-bib-0013] Camacho Carvajal, M. M. , A. H. M. Wijfjes , I. H. M. Mulders , B. J. J. Lugtenberg , and G. V. Bloemberg . 2002. “Characterization of NADH Dehydrogenases of *Pseudomonas fluorescens* WCS365 and Their Role in Competitive Root Colonization.” Molecular Plant‐Microbe Interactions 15: 662–671. 12118882 10.1094/MPMI.2002.15.7.662

[emi470179-bib-0014] Canova, M. J. , and V. Molle . 2014. “Bacterial Serine/Threonine Protein Kinases in Host‐Pathogen Interactions.” Journal of Biological Chemistry 289: 9473–9479. 24554701 10.1074/jbc.R113.529917 PMC3974997

[emi470179-bib-0015] Cantalapiedra, C. P. , A. Hernández‐Plaza , I. Letunic , P. Bork , and J. Huerta‐Cepas . 2021. “eggNOG‐Mapper v2: Functional Annotation, Orthology Assignments, and Domain Prediction at the Metagenomic Scale.” Molecular Biology and Evolution 38: 5825–5829. 34597405 10.1093/molbev/msab293 PMC8662613

[emi470179-bib-0016] Chevalier, S. , E. Bouffartigues , J. Bodilis , et al. 2017. “Structure, Function and Regulation of *Pseudomonas aeruginosa* Porins.” FEMS Microbiology Reviews 41: 698–722. 28981745 10.1093/femsre/fux020

[emi470179-bib-0017] Cole, B. J. , M. E. Feltcher , R. J. Waters , et al. 2017. “Genome‐Wide Identification of Bacterial Plant Colonization Genes.” PLoS Biology 15: e2002860. 28938018 10.1371/journal.pbio.2002860 PMC5627942

[emi470179-bib-0018] Consortium . 2022. “UniProt: The Universal Protein Knowledgebase in 2023.” Nucleic Acids Research 51: D523–D531. 10.1093/nar/gkac1052 PMC9825514 36408920

[emi470179-bib-0019] D'aes, J. , N. P. Kieu , V. Léclère , et al. 2014. “To Settle or to Move? The Interplay Between Two Classes of Cyclic Lipopeptides in the Biocontrol Strain *Pseudomonas* CMR12a.” Environmental Microbiology 16: 2282–2300. 24673852 10.1111/1462-2920.12462

[emi470179-bib-0020] Dailey, F. E. , and H. C. Berg . 1993. “Mutants in Disulfide Bond Formation That Disrupt Flagellar Assembly in *Escherichia coli* .” Proceedings of the National Academy of Sciences 90: 1043–1047. 10.1073/pnas.90.3.1043 PMC45807 8503954

[emi470179-bib-0021] de Vries, F. T. , R. I. Griffiths , C. G. Knight , O. Nicolitch , and A. Williams . 2020. “Harnessing Rhizosphere Microbiomes for Drought‐Resilient Crop Production.” Science 368: 270–274. 32299947 10.1126/science.aaz5192

[emi470179-bib-0022] Dubern, J.‐F. , E. L. Lagendijk , B. J. J. Lugtenberg , and G. V. Bloemberg . 2005. “The Heat Shock Genes *dnaK*, *dnaJ*, and *grpE* Are Involved in Regulation of Putisolvin Biosynthesis in *Pseudomonas Putida* PCL1445.” Journal of Bacteriology 187: 5967–5976. 16109938 10.1128/JB.187.17.5967-5976.2005 PMC1196155

[emi470179-bib-0023] Emms, D. M. , and S. Kelly . 2019. “OrthoFinder: Phylogenetic Orthology Inference for Comparative Genomics.” Genome Biology 20: 238. 31727128 10.1186/s13059-019-1832-y PMC6857279

[emi470179-bib-0024] Fabian, B. K. , C. Foster , A. Asher , K. A. Hassan , I. T. Paulsen , and S. G. Tetu . 2024. “Identifying the Suite of Genes Central to Swimming in the Biocontrol Bacterium *Pseudomonas protegens* pf‐5.” Microbial Genomics 10: 1212. 10.1099/mgen.1090.001212. PMC11004494 38546328

[emi470179-bib-0025] Fabian, B. K. , C. Foster , A. J. Asher , et al. 2021. “Elucidating Essential Genes in Plant‐Associated *Pseudomonas protegens* pf‐5 Using Transposon Insertion Sequencing.” Journal of Bacteriology 203: 00420. 10.1128/JB.00432-20 PMC8088517 33257523

[emi470179-bib-0026] Gross, H. , V. O. Stockwell , M. D. Henkels , B. Nowak‐Thompson , J. E. Loper , and W. H. Gerwick . 2007. “The Genomisotopic Approach: A Systematic Method to Isolate Products of Orphan Biosynthetic Gene Clusters.” Chemistry and Biology 14: 53–63. 17254952 10.1016/j.chembiol.2006.11.007

[emi470179-bib-0027] Hernández, L. , D. Afonso , E. M. Rodríguez , and C. Díaz . 2011. “Phenolic Compounds in Wheat Grain Cultivars.” Plant Foods for Human Nutrition 66: 408–415. 22038325 10.1007/s11130-011-0261-1

[emi470179-bib-0028] Hoagland, D. R. , and D. I. Arnon . 1938. “The Water‐Culture Method for Growing Plants Without Soil.” California Agricultural Experiment Station: Circular‐347.

[emi470179-bib-0029] Hori, K. , and S. Matsumoto . 2010. “Bacterial Adhesion: From Mechanism to Control.” Biochemical Engineering Journal 48: 424–434.

[emi470179-bib-0030] Howell, C. , and R. Stipanovic . 1979. “Control of *Rhizoctonia solani* on Cotton Seedlings With *Pseudomonas fluorescens* and With an Antibiotic Produced by the Bacterium.” Phytopathology 69: 480–482. 10.1094/Phyto-69-480 40934404

[emi470179-bib-0031] Howell, C. , and R. Stipanovic . 1980. “Suppression of *Pythium ultimum*‐Induced Damping‐Off of Cotton Seedlings by *Pseudomonas fluorescens* and Its Antibiotic, Pyoluteorin.” Phytopathology 70: 712–715.

[emi470179-bib-0032] Huerta‐Cepas, J. , D. Szklarczyk , D. Heller , et al. 2018. “eggNOG 5.0: A Hierarchical, Functionally and Phylogenetically Annotated Orthology Resource Based on 5090 Organisms and 2502 Viruses.” Nucleic Acids Research 47, no. D1: D309–D314. 10.1093/nar/gky1085 PMC6324079 30418610

[emi470179-bib-0033] Jorgenson, M. A. , Y. Chen , A. Yahashiri , D. L. Popham , and D. S. Weiss . 2014. “The Bacterial Septal Ring Protein RlpA Is a Lytic Transglycosylase That Contributes to Rod Shape and Daughter Cell Separation in Seudomonas Aeruginosa.” Molecular Microbiology 93: 113–128. 24806796 10.1111/mmi.12643 PMC4086221

[emi470179-bib-0034] Kanemaru, Y. , F. Hasebe , T. Tomita , T. Kuzuyama , and M. Nishiyama . 2013. “Two ATP‐Binding Cassette Transporters Involved in (*S*)‐2‐Aminoethyl‐Cysteine Uptake in *Thermus thermophilus* .” Journal of Bacteriology 195: 3845–3853. 23794618 10.1128/JB.00202-13 PMC3754599

[emi470179-bib-0035] Keegstra, J. M. , F. Carrara , and R. Stocker . 2022. “The Ecological Roles of Bacterial Chemotaxis.” Nature Reviews. Microbiology 20: 491–504. 35292761 10.1038/s41579-022-00709-w

[emi470179-bib-0036] Kinscherf, T. G. , and D. K. Willis . 2002. “Global Regulation by *gidA* in *Pseudomonas Syringae* .” Journal of Bacteriology 184: 2281–2286. 11914360 10.1128/JB.184.8.2281-2286.2002 PMC134964

[emi470179-bib-0037] Klementiev, A. D. , Z. Jin , and M. Whiteley . 2020. “Micron Scale Spatial Measurement of the O_2_ Gradient Surrounding a Bacterial Biofilm in Real Time.” MBio 11: 10–1128. 10.1128/mbio.02536-02520. PMC7587442 33082251

[emi470179-bib-0038] Kloek, A. P. , D. M. Brooks , and B. N. Kunkel . 2000. “A *dsbA* Mutant of *Pseudomonas Syringae* Exhibits Reduced Virulence and Partial Impairment of Type III Secretion.” Molecular Plant Pathology 1: 139–150. 20572960 10.1046/j.1364-3703.2000.00016.x

[emi470179-bib-0039] Kouakou, T. H. , P. Waffo‐Téguo , Y. J. Kouadio , et al. 2007. “Phenolic Compounds and Somatic Embryogenesis in Cotton ( *Gossypium hirsutum* L.).” Plant Cell, Tissue and Organ Culture 90: 25–29.

[emi470179-bib-0040] Kraus, J. , and J. E. Loper . 1992. “Lack of Evidence for a Role of Antifungal Metabolite Production by *Pseudomonas Fluorescens* pf‐5 in Biological Control of *Pythium* Damping‐Off of Cucumber.” Phytopathology 82: 264–271.

[emi470179-bib-0041] Krueger, J. , M. Preusse , N. Oswaldo Gomez , et al. 2024. “tRNA Epitranscriptome Determines Pathogenicity of the Opportunistic Pathogen *Pseudomonas aeruginosa* .” Proceedings of the National Academy of Sciences of the United States of America 121: e2312874121. 38451943 10.1073/pnas.2312874121 PMC10945773

[emi470179-bib-0042] Kuiper, I. , G. V. Bloemberg , S. Noreen , J. E. Thomas‐Oates , and B. J. J. Lugtenberg . 2001. “Increased Uptake of Putrescine in the Rhizosphere Inhibits Competitive Root Colonization by *Pseudomonas fluorescens* Strain WCS365.” Molecular Plant‐Microbe Interactions 14: 1096–1104. 11551074 10.1094/MPMI.2001.14.9.1096

[emi470179-bib-0043] Kumar, R. , R. Bhatia , K. Kukreja , R. K. Behl , S. S. Dudeja , and N. Narula . 2007. “Establishment of *Azotobacter* on Plant Roots: Chemotactic Response, Development and Analysis of Root Exudates of Cotton (*Gossypium hirsutum* L.) and Wheat (*Triticum aestivum* L.).” Journal of Basic Microbiology 47: 436–439. 17910096 10.1002/jobm.200610285

[emi470179-bib-0044] Langridge, G. C. , M.‐D. Phan , D. J. Turner , et al. 2009. “Simultaneous Assay of Every *Salmonella typhi* Gene Using One Million Transposon Mutants.” Genome Research 19: 2308–2316. 19826075 10.1101/gr.097097.109 PMC2792183

[emi470179-bib-0045] Lecomte, S. M. , W. Achouak , D. Abrouk , T. Heulin , X. Nesme , and F. E. Z. Haichar . 2018. “Diversifying Anaerobic Respiration Strategies to Compete in the Rhizosphere.” Frontiers in Environmental Science 6: 139.

[emi470179-bib-0046] Lemanceau, P. , P. Bauer , S. Kraemer , and J.‐F. Briat . 2009. “Iron Dynamics in the Rhizosphere as a Case Study for Analyzing Interactions Between Soils, Plants and Microbes.” Plant and Soil 321: 513–535.

[emi470179-bib-0047] Losensky, G. , K. Jung , H. Urlaub , F. Pfeifer , S. Fröls , and C. Lenz . 2017. “Shedding Light on Biofilm Formation of *Halobacterium salinarum* R1 by SWATH‐LC/MS/MS Analysis of Planktonic and Sessile Cells.” Proteomics 17: 1600111. 10.1002/pmic.201600111 27604596

[emi470179-bib-0048] Lu, H. , Y. Que , X. Wu , T. Guan , and H. Guo . 2019. “Metabolomics Deciphered Metabolic Reprogramming Required for Biofilm Formation.” Scientific Reports 9: 13160. 31511592 10.1038/s41598-019-49603-1 PMC6739361

[emi470179-bib-0049] Lugtenberg, B. J. J. , L. Dekkers , and G. V. Bloemberg . 2001. “Molecular Determinants of Rhizosphere Colonization.” Annual Review of Phytopathology 39: 461–490. 10.1146/annurev.phyto.39.1.461 11701873

[emi470179-bib-0050] Ma, Z. , N. Geudens , N. P. Kieu , et al. 2016. “Biosynthesis, Chemical Structure, and Structure‐Activity Relationship of Orfamide Lipopeptides Produced by *Pseudomonas protegens* and Related Species.” Frontiers in Microbiology 7: 382. 27065956 10.3389/fmicb.2016.00382 PMC4811929

[emi470179-bib-0051] Mavrodi, O. V. , D. V. Mavrodi , A. A. Park , D. M. Weller , and L. S. Thomashow . 2006. “The Role of *dsbA* in Colonization of the Wheat Rhizosphere by *Pseudomonas fluorescens* Q8r1‐96.” Microbiology 152: 863–872. 16514165 10.1099/mic.0.28545-0

[emi470179-bib-0052] McDonald, H. J. , H. Kweon , S. Kurnfuli , and D. D. Risser . 2022. “A DnaK(Hsp70) Chaperone System Connects Type IV Pilus Activity to Polysaccharide Secretion in Cyanobacteria.” MBio 13: e00514‐00522. 35420478 10.1128/mbio.00514-22 PMC9239167

[emi470179-bib-0053] McNeil, M. B. , H. G. Hampton , K. J. Hards , B. N. J. Watson , G. M. Cook , and P. C. Fineran . 2014. “The Succinate Dehydrogenase Assembly Factor, SdhE, Is Required for the Flavinylation and Activation of Fumarate Reductase in Bacteria.” FEBS Letters 588: 414–421. 24374335 10.1016/j.febslet.2013.12.019

[emi470179-bib-0054] Meyer, S. , A. Scrima , W. Versées , and A. Wittinghofer . 2008. “Crystal Structures of the Conserved tRNA‐Modifying Enzyme GidA: Implications for Its Interaction With MnmE and Substrate.” Journal of Molecular Biology 380: 532–547. 18565343 10.1016/j.jmb.2008.04.072

[emi470179-bib-0055] Mohammed, M. M. A. , V. K. Pettersen , A. H. Nerland , H. G. Wiker , and V. Bakken . 2017. “Quantitative Proteomic Analysis of Extracellular Matrix Extracted From Mono‐And Dual‐Species Biofilms of *Fusobacterium nucleatum* and *Porphyromonas gingivalis* .” Anaerobe 44: 133–142. 28285095 10.1016/j.anaerobe.2017.03.002

[emi470179-bib-0056] Morinière, L. , L. Mirabel , E. Gueguen , and F. Bertolla . 2022. “A Comprehensive Overview of the Genes and Functions Required for Lettuce Infection by the Hemibiotrophic Phytopathogen *Xanthomonas Hortorum* Pv. Vitians.” Msystems 7: e01290‐01221. 35311560 10.1128/msystems.01290-21 PMC9040725

[emi470179-bib-0057] Nielsen, T. H. , O. Nybroe , B. Koch , M. Hansen , and J. Sørensen . 2005. “Genes Involved in Cyclic Lipopeptide Production Are Important for Seed and Straw Colonization by *Pseudomonas* sp. Strain DSS73.” Applied and Environmental Microbiology 71: 4112–4116. 16000829 10.1128/AEM.71.7.4112-4116.2005 PMC1169020

[emi470179-bib-0058] O'Callaghan, M. , R. A. Ballard , and D. Wright . 2022. “Soil Microbial Inoculants for Sustainable Agriculture: Limitations and Opportunities.” Soil Use and Management 38: 1340–1369.

[emi470179-bib-0059] Pan, X. , H. Zhang , Y. Gao , M. Li , and W. Chang . 2009. “Crystal Structures of *Pseudomonas syringae* Pv. Tomato DC3000 Quinone Oxidoreductase and Its Complex With NADPH.” Biochemical and Biophysical Research Communications 390: 597–602. 19818736 10.1016/j.bbrc.2009.10.012

[emi470179-bib-0060] Paulsen, I. T. , C. M. Press , J. Ravel , et al. 2005. “Complete Genome Sequence of the Plant Commensal *Pseudomonas fluorescens* pf‐5.” Nature Biotechnology 23: 873–878. 10.1038/nbt1110 PMC7416659 15980861

[emi470179-bib-0061] Pfender, W. F. , J. Kraus , and J. E. Loper . 1993. “A Genomic Region From *Pseudomonas fluorescens* pf‐5 Required for Pyrrolnitrin Production and Inhibition of *Pyrenophora Tritici‐Repentis* in Wheat Straw.” Phytopathology 83: 1223–1228.

[emi470179-bib-0062] Pliego, C. , J. I. Crespo‐Gómez , A. Pintado , et al. 2019. “Response of the Biocontrol Agent *Pseudomonas pseudoalcaligenes* AVO110 to *Rosellinia necatrix* Exudate.” Applied and Environmental Microbiology 85: e01741. 30478234 10.1128/AEM.01741-18 PMC6344628

[emi470179-bib-0063] Price, M. N. , A. M. Deutschbauer , and A. P. Arkin . 2020. “GapMind: Automated Annotation of Amino Acid Biosynthesis.” MSystems 5: 10–1128. 10.1128/msystems.00291-00220. PMC7311316 32576650

[emi470179-bib-0064] Qin, H. , Y. Liu , X. Cao , et al. 2020. “RpoS Is a Pleiotropic Regulator of Motility, Biofilm Formation, Exoenzymes, Siderophore and Prodigiosin Production, and Trade‐Off During Prolonged Stationary Phase in *Serratia marcescens* .” PLoS One 15: e0232549. 32484808 10.1371/journal.pone.0232549 PMC7266296

[emi470179-bib-0065] Robinson, M. D. , D. J. McCarthy , and G. K. Smyth . 2010. “edgeR: A Bioconductor Package for Differential Expression Analysis of Digital Gene Expression Data.” Bioinformatics 26: 139–140. 19910308 10.1093/bioinformatics/btp616 PMC2796818

[emi470179-bib-0066] Rodríguez‐Martínez, K. , L. F. Muriel‐Millán , C. Ortíz‐Vasco , S. Moreno , G. Soberón‐Chávez , and G. Espín . 2023. “Defining the Regulatory Mechanisms of Sigma Factor RpoS Degradation in *Azotobacter vinelandii* and *Pseudomonas aeruginosa* .” Molecular Microbiology 120: 91–102. 37328957 10.1111/mmi.15107

[emi470179-bib-0067] Saravanan, S. , P. Muthumanickam , T. Saravanan , and K. Santhaguru . 2013. “Antagonistic Potential of Fluorescent *Pseudomonas* and Its Impact on Growth of Tomato Challenged With Phtopathogens.” African Crop Science Journal 21: 29–36.

[emi470179-bib-0068] Shahwar, D. , Z. Mushtaq , H. Mushtaq , et al. 2023. “Role of Microbial Inoculants as Bio Fertilizers for Improving Crop Productivity: A Review.” Heliyon 9: e16134. 37255980 10.1016/j.heliyon.2023.e16134 PMC10225898

[emi470179-bib-0069] Shevchik, V. E. , I. Bortoli‐Gernnan , J. Robert‐Baudouy , S. Robinet , F. Barras , and G. Condemine . 1995. “Differential Effect of *dsbA* and *dsbC* Mutations on Extracellular Enzyme Secretion in *Erwinia chrysanthemi* .” Molecular Microbiology 16: 745–753. 7476168 10.1111/j.1365-2958.1995.tb02435.x

[emi470179-bib-0070] Silby, M. W. , A. M. Cerdeño‐Tárraga , G. S. Vernikos , et al. 2009. “Genomic and Genetic Analyses of Diversity and Plant Interactions of *Pseudomonas fluorescens* .” Genome Biology 10: R51. 19432983 10.1186/gb-2009-10-5-r51 PMC2718517

[emi470179-bib-0071] Simons, M. , H. P. Permentier , L. A. de Weger , C. A. Wijffelman , and B. J. J. Lugtenberg . 1997. “Amino Acid Synthesis Is Necessary for Tomato Root Colonization by *Pseudomonas fluorescens* Strain WCS365.” Molecular Plant‐Microbe Interactions 10: 102–106.

[emi470179-bib-0072] Sinha, A. K. , A. Durand , J. M. Desfontaines , et al. 2017. “Division‐Induced DNA Double Strand Breaks in the Chromosome Terminus Region of *Escherichia coli* Lacking RecBCD DNA Repair Enzyme.” PLoS Genetics 13: e1006895. 28968392 10.1371/journal.pgen.1006895 PMC5638614

[emi470179-bib-0073] Sivakumar, R. , P. Gunasekaran , and J. Rajendhran . 2021. “Inactivation of CbrAB Two‐Component System Hampers Root Colonization in Rhizospheric Strain of *Pseudomonas aeruginosa* PGPR2.” Biochimica et Biophysica Acta 1864: 194763. 34530138 10.1016/j.bbagrm.2021.194763

[emi470179-bib-0074] Sivakumar, R. , J. Ranjani , U. S. Vishnu , et al. 2019. “Evaluation of INSeq to Identify Genes Essential for *Pseudomonas aeruginosa* PGPR2 Corn Root Colonization.” G3: Genes, Genomes, Genetics 9, no. 3: 651–661. 30705119 10.1534/g3.118.200928 PMC6404608

[emi470179-bib-0075] Song, C. , G. Sundqvist , E. Malm , et al. 2015. “Lipopeptide Biosynthesis in *Pseudomonas fluorescens* Is Regulated by the Protease Complex ClpAP.” BMC Microbiology 15: 29. 25885431 10.1186/s12866-015-0367-y PMC4332742

[emi470179-bib-0076] Srimahaeak, T. , N. Thongdee , J. Chittrakanwong , et al. 2023. “ *Pseudomonas aeruginosa* GidA Modulates the Expression of Catalases at the Posttranscriptional Level and Plays a Role in Virulence.” Frontiers in Microbiology 13: 1079710. 36726575 10.3389/fmicb.2022.1079710 PMC9884967

[emi470179-bib-0077] Sriramulu, D. D. , H. Lünsdorf , J. S. Lam , and U. Römling . 2005. “Microcolony Formation: A Novel Biofilm Model of *Pseudomonas aeruginosa* for the Cystic Fibrosis Lung.” Journal of Medical Microbiology 54: 667–676. 15947432 10.1099/jmm.0.45969-0

[emi470179-bib-0078] Stewart, P. S. , T. Zhang , R. Xu , et al. 2016. “Reaction–Diffusion Theory Explains Hypoxia and Heterogeneous Growth Within Microbial Biofilms Associated With Chronic Infections.” Npj Biofilms and Microbiomes 2: 16012. 28721248 10.1038/npjbiofilms.2016.12 PMC5515263

[emi470179-bib-0079] Sun, J. , F. Lu , Y. Luo , L. Bie , L. Xu , and Y. Wang . 2023. “OrthoVenn3: An Integrated Platform for Exploring and Visualizing Orthologous Data Across Genomes.” Nucleic Acids Research 51: W397–W403. 37114999 10.1093/nar/gkad313 PMC10320085

[emi470179-bib-0080] Suryaletha, K. , L. Narendrakumar , J. John , M. P. Radhakrishnan , S. George , and S. Thomas . 2019. “Decoding the Proteomic Changes Involved in the Biofilm Formation of *Enterococcus faecalis* SK460 to Elucidate Potential Biofilm Determinants.” BMC Microbiology 19: 146. 31253082 10.1186/s12866-019-1527-2 PMC6599329

[emi470179-bib-0081] Torres, A. , N. Kasturiarachi , M. DuPont , V. S. Cooper , J. Bomberger , and A. Zemke . 2019. “NADH Dehydrogenases in *Pseudomonas aeruginosa* Growth and Virulence.” Frontiers in Microbiology 10: 75. 30804898 10.3389/fmicb.2019.00075 PMC6370648

[emi470179-bib-0082] Torres‐Sánchez, L. , T. G. Sana , M. De Cossas , Y. Hashem , and P. V. Krasteva . 2023. “Structures of the *P. aeruginosa* FleQ‐FleN Master Regulators Reveal Large‐Scale Conformational Switching in Motility and Biofilm Control.” Proceedings of the National Academy of Sciences of the United States of America 120: e2312276120. 38051770 10.1073/pnas.2312276120 PMC10723142

[emi470179-bib-0083] Tran, H. , A. Ficke , T. Asiimwe , M. Höfte , and J. M. Raaijmakers . 2007. “Role of the Cyclic Lipopeptide Massetolide A in Biological Control of *Phytophthora infestans* and in Colonization of Tomato Plants by *Pseudomonas fluorescens* .” New Phytologist 175: 731–742. 17688588 10.1111/j.1469-8137.2007.02138.x

[emi470179-bib-0084] Trivedi, P. , J. E. Leach , S. G. Tringe , T. Sa , and B. K. Singh . 2020. “Plant–Microbiome Interactions: From Community Assembly to Plant Health.” Nature Reviews. Microbiology 18: 607–621. 32788714 10.1038/s41579-020-0412-1

[emi470179-bib-0085] Van Dongen, S. 2000. Graph Clustering by Flow Simulation. University of Utrecht.

[emi470179-bib-0086] van Kempen, M. , S. S. Kim , C. Tumescheit , et al. 2024. “Fast and Accurate Protein Structure Search With Foldseek.” Nature Biotechnology 42: 243–246. 10.1038/s41587-023-01773-0 PMC10869269 37156916

[emi470179-bib-0087] Varadi, M. , D. Bertoni , P. Magana , et al. 2023. “AlphaFold Protein Structure Database in 2024: Providing Structure Coverage for Over 214 Million Protein Sequences.” Nucleic Acids Research 52, no. D1: D368–D375. 10.1093/nar/gkad1011 PMC10767828 37933859

[emi470179-bib-0088] Vick, S. H. W. , B. K. Fabian , C. J. Dawson , et al. 2021. “Delving Into Defence: Identifying the *Pseudomonas protegens* pf‐5 Gene Suite Involved in Defence Against Secreted Products of Fungal, Oomycete and Bacterial Rhizosphere Competitors.” Microbial Genomics 7: 000671. 34788213 10.1099/mgen.0.000671 PMC8743541

[emi470179-bib-0089] Walker, T. S. , H. P. Bais , E. Grotewold , and J. M. Vivanco . 2003. “Root Exudation and Rhizosphere Biology.” Plant Physiology 132: 44–51. 12746510 10.1104/pp.102.019661 PMC1540314

[emi470179-bib-0090] Wei, J. R. , and H. C. Lai . 2006. “ *N*‐Acylhomoserine Lactone‐Dependent Cell‐To‐Cell Communication and Social Behavior in the Genus *Serratia* .” International Journal of Medical Microbiology 296: 117–124. 16483841 10.1016/j.ijmm.2006.01.033

[emi470179-bib-0091] Xu, G. , and D. Gross . 1986. “Selection of Fluorescent Pseudomonads Antagonistic to *Erwinia carotovora* and Suppressive of Potato Seed Piece Decay.” Phytopathology 76: 414–422.

[emi470179-bib-0092] Yoshioka, S. , and P. D. Newell . 2016. “Disruption of De Novo Purine Biosynthesis in *Pseudomonas fluorescens* Pf0‐1 Leads to Reduced Biofilm Formation and a Reduction in Cell Size of Surface‐Attached but Not Planktonic Cells.” PeerJ 4: e1543. 26788425 10.7717/peerj.1543 PMC4715448

[emi470179-bib-0093] Zboralski, A. , and M. Filion . 2020. “Genetic Factors Involved in Rhizosphere Colonization by Phytobeneficial *Pseudomonas* Spp.” Computational and Structural Biotechnology Journal 18: 3539–3554. 33304453 10.1016/j.csbj.2020.11.025 PMC7711191

