# Supplementary material for: Genetic Drivers of Plant Root Colonisation by the Biocontrol Agent Pseudomonas protegens Pf‐5
Source: Environ Microbiol Rep. 2025 Aug 19;17(4):e70179. doi: 10.1111/1758-2229.70179 (PMC12361813; doi:10.1111/1758-2229.70179)
Supplement: Supplementary file 1 — Figure S1. Pseudomonas protegens Pf‐5 root colonisation and TraDIS duplicate reproducibility. (a) Scanning electron microscopy images of Pf‐5 colonisation of cotton roots (top) and wheat roots (bottom). (b) Correlation of gene insertion indexes for the duplicate TraDIS assays for cotton (top) and wheat (bottom). Insertion indices represent the number of transposon insertion sites for a given gene normalised by gene length. Figure S2. Differential frequencies of all Pseudomonas protegens Pf‐5 gene insertions. Volcano plots show log2 fold change and −log10 q‐values for root colonisation assays of a, cotton, and b, wheat, relative to the control. Points in dark blue have a significant log2‐fold change < −2, or > 2, and a q‐value < 0.01. [file EMI4-17-e70179-s002.docx]

Supplementary Information for

**Identifying the *Pseudomonas protegens* Pf-5 genes involved in plant root colonisation**

Timothy M. Ghaly, Belinda K. Fabian, Silas H. W. Vick, Christie Foster, Amy J. Asher, Karl A. Hassan, Liam D. H. Elbourne, Ian T. Paulsen, Sasha G. Tetu

**The PDF file includes:**

Supplementary Figs. S1 and S2

**Other Supplementary Information for this manuscript include the following:**

Supplementary Tables S1-S4


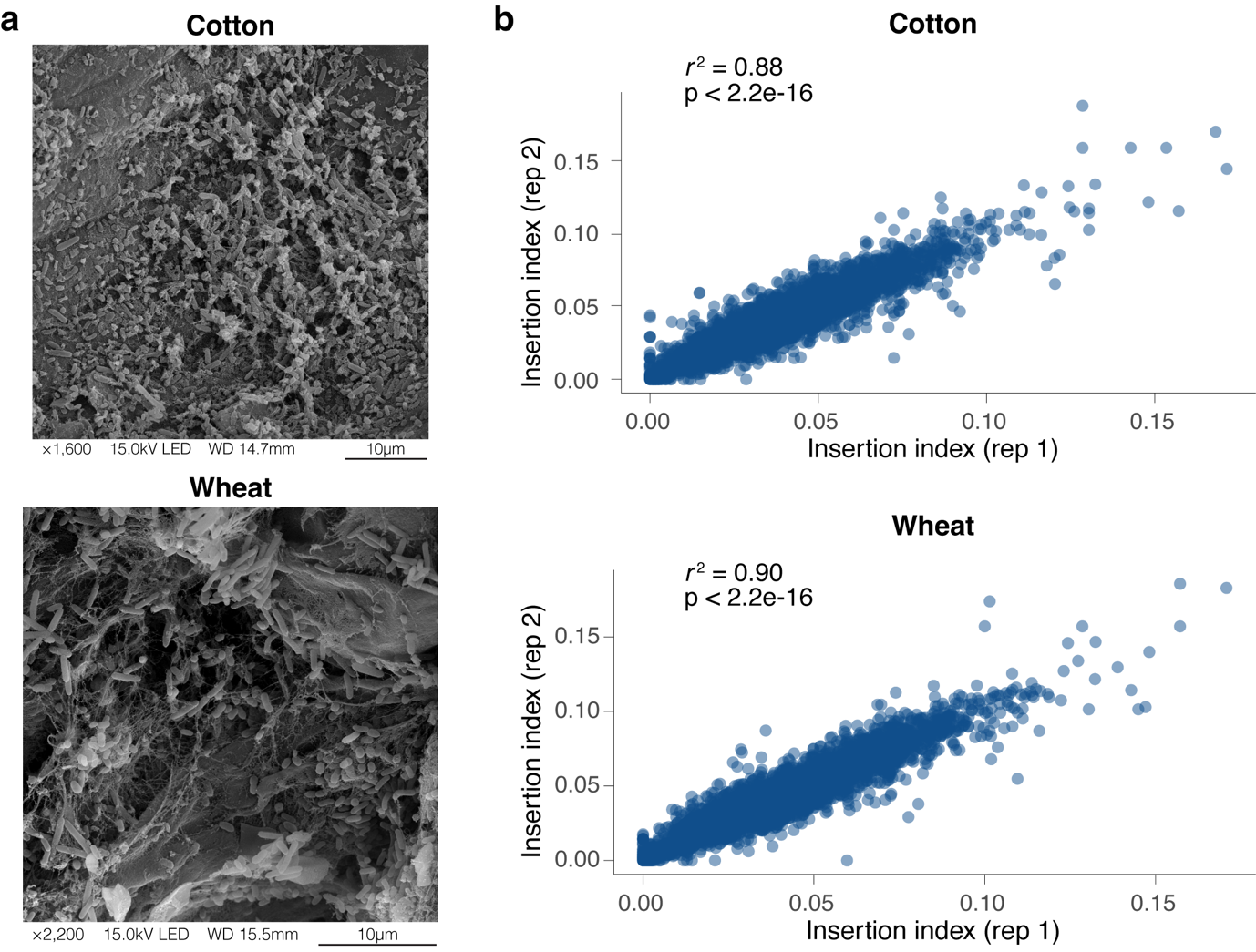


**Supplementary Fig. S1. *Pseudomonas protegens* Pf-5 root colonisation and TraDIS duplicate reproducibility. a**, Scanning electron microscopy images of Pf-5 colonisation of cotton roots (top) and wheat roots (bottom). **b**, Correlation of gene insertion indexes for the duplicate TraDIS assays for cotton (top) and wheat (bottom). Insertion indices represent the number of transposon insertion sites for a given gene normalised by gene length.


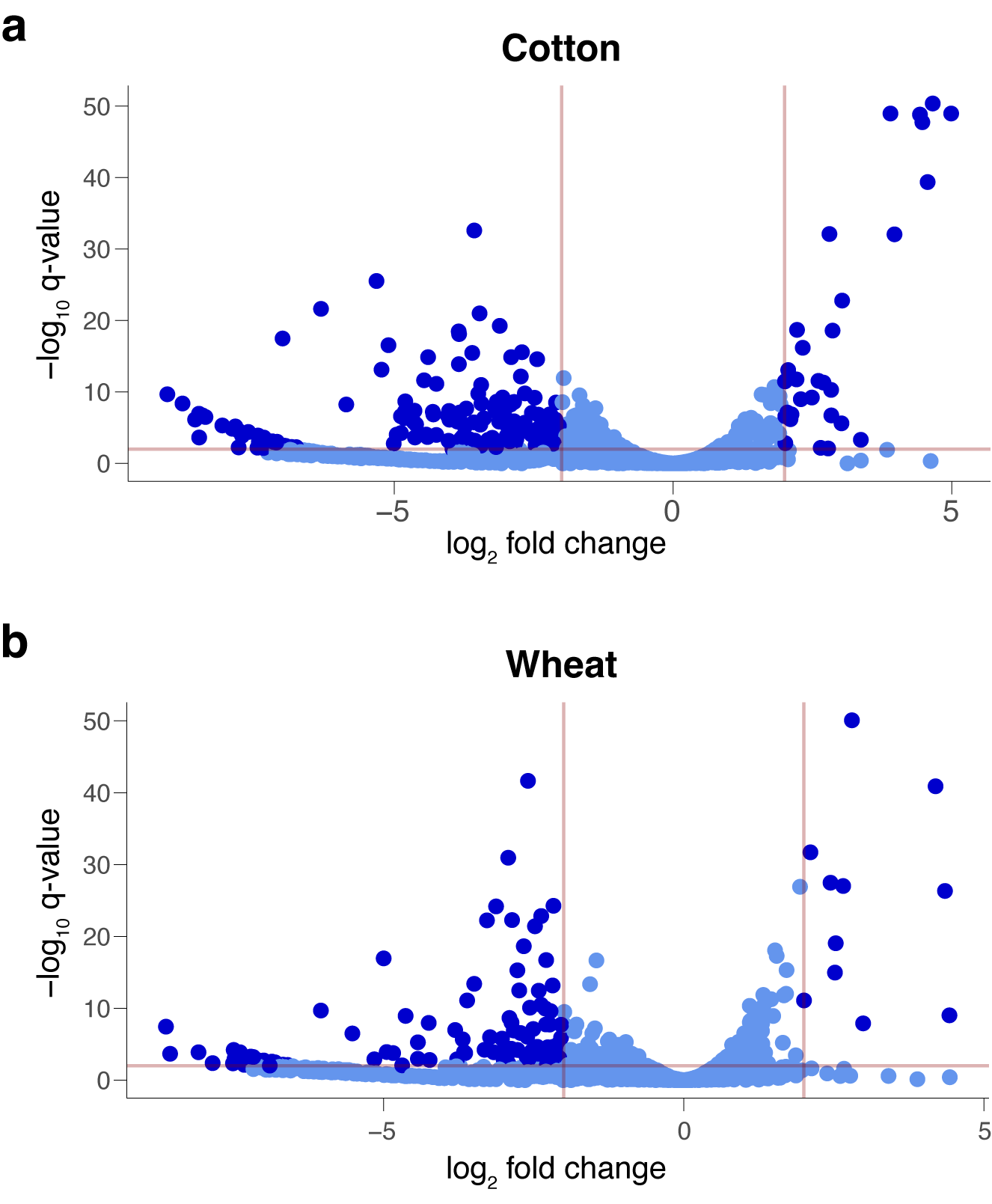


**Supplementary Fig. S2. Differential frequencies of all *Pseudomonas protegens* Pf-5 gene insertions.** Volcano plots show log_2_ fold change and -log_10_ q-values for root colonisation assays of **a**, cotton, and **b**, wheat, relative to the control. Points in dark blue have a significant log_2_-fold change < −2, or > 2, and a q-value < 0.01.
